# Association between hypovitaminosis D and frequency of pulmonary exacerbations in children and adolescents with cystic fibrosis

**DOI:** 10.1590/S1679-45082018AO4143

**Published:** 2018-04-19

**Authors:** Renata Ongaratto, Katiana Murieli da Rosa, Juliana Cristina Eloi, Matias Epifanio, Paulo Marostica, Leonardo Araújo Pinto

**Affiliations:** 1 Pontifícia Universidade Católica do Rio Grande do Sul, Porto Alegre, RS, Brazil; 2 Hospital São Lucas, Pontifícia Universidade Católica do Rio Grande do Sul, Porto Alegre, RS, Brazil; 3 Universidade Federal do Rio Grande do Sul, Porto Alegre, RS, Brazil

**Keywords:** Vitamin D, Avitaminosis, Cystic fibrosis, Symptom flare up, Child, Adolescent, Vitamina D, Deficiência de vitaminas, Fibrose cística, Exacerbação dos sintomas, Criança, Adolescente

## Abstract

**Objective:**

We evaluated the association between vitamin D levels and nutritional status, pulmonary function and pulmonary exacerbations in children and adolescents with cystic fibrosis.

**Methods:**

25-hydroxyvitamin D (25(OH)D) levels of 37 children and adolescents were retrospectively evaluated. Pulmonary function, body mass index, height for age, and pulmonary exacerbations episodes were associated with vitamin D levels divided into two groups: sufficient (≥30ng/mL) and hypovitaminosis (<30ng/mL).

**Results:**

Hypovitaminosis D (25(OH)D <30ng/mL) was observed in 54% of subjects. The mean level of 25(OH)D was 30.53±12.14ng/mL. Pulmonary function and nutritional status were not associated with vitamin D levels. Pulmonary exacerbations over a 2-year period (p=0.007) and the period from measurement up to the end of the follow-up period (p=0.002) were significantly associated with vitamin D levels.

**Conclusion:**

Hypovitaminosis D was associated with higher rates of pulmonary exacerbations in this sample of children and adolescents with cystic fibrosis. Hypovitaminosis D should be further studied as a marker of disease severity in cystic fibrosis. Further prospective and randomized studies are necessary to investigate causality of this association.

## RESUMO

**Objetivo:**

Avaliar a associação entre níveis de vitamina D e estado nutricional, função pulmonar e exacerbações pulmonares em crianças e adolescentes com fibrose cística.

**Métodos:**

Os níveis de 25-hidroxivitamina D (25(OH)D) de 37 crianças e adolescentes foram avaliados retrospectivamente. Função pulmonar, índice de massa corporal, altura para a idade, e episódios de exacerbações pulmonares foram associados aos níveis de vitamina D divididos em dois grupos: suficiente (≥30ng/mL) e hipovitaminose (<30ng/mL).

**Resultados:**

Hipovitaminose D (25(OH)D <30ng/mL) foi observada em 54% dos pacientes. O nível médio de 25(OH)D foi de 30,53±12,14ng/mL. A função pulmonar e o estado nutricional não foram associados aos níveis de vitamina D. As exacerbações pulmonares durante um período de 2 anos (p = 0,007) e do período de medição até o final do período de seguimento (p=0,002) foram significativamente associadas aos níveis de vitamina D.

**Conclusão:**

A hipovitaminose D esteve associada a maiores taxas de exacerbações pulmonares nesta amostra de crianças e adolescentes com fibrose cística. A hipovitaminose D deve ser mais estudada como marcador da gravidade da doença na fibrose cística. Outros estudos prospectivos e randomizados são necessários para investigar a relação causal desta associação.

## INTRODUCTION

Vitamin D is recognized for its role in bone mineralization and calcium homeostasis.^(^ ^1^ ^)^ Current evidence has shown extra skeletal functions of vitamin D.^(^ ^2^ ^,^ ^3^ ^)^ Some studies have suggested a potential benefit of vitamin D on respiratory health, although the mechanisms have not yet been well understood.^(^ ^4^ ^–^ ^7^ ^)^

Several disorders have been associated with an increased risk of vitamin D deficiency, especially those with malabsorption, such as cystic fibrosis (CF).^(^ ^3^ ^,^ ^8^ ^)^Cystic fibrosis is the most common inherited respiratory disease in the Western world, with an estimated incidence of 1/3,000 to 1/9,000 live births.^(^ ^8^ ^)^ The Brazilian Cystic Fibrosis Registry (2013) reached 2,669 subjects, with 94% being followed up at CF centers countrywide.^(^ ^9^ ^)^Cystic fibrosis is associated with progressive lung disease, pancreatic insufficienc y and malnutrition, which are considered major determinants of prognosis.^(^ ^10^ ^)^Pulmonary exacerbations (PE) are a major cause of morbidity and decreased the quality of life of CF subjects. Furthermore, recurrent and frequent PE have been linked to decreased survival.^(^ ^11^ ^,^ ^12^ ^)^

Suboptimal levels of vitamin D in subjects with CF are common^(^ ^13^ ^–^ ^16^ ^)^ and probably result from a combination of factors, such as inadequate absorption, impaired metabolism and lack of sun exposure. Hypovitaminosis D in CF has been associated with an increased predisposition to bone disease and impaired pulmonary function (PF).^(^ ^10^ ^,^ ^17^ ^)^ There are only a few studies linking vitamin D with lung function or PE in CF children and adolescents. Thus, further research regarding the implications of vitamin D deficiency in CF clinical outcomes is needed.

## OBJECTIVE

To investigate vitamin D levels of cystic fibrosis subjects and their association with nutritional status, pulmonary function and pulmonary exacerbations.

## METHODS

This is a retrospective cross-sectional study. The population consisted of CF subjects aged 1 to 20 years, followed up at a CF center in Southern Brazil. From 80 registered subjects, the study enrolled all subjects who had 25(OH)D measurements available. When subjects had more than one 25(OH)D measurement, we registered all of them and considered the most recent one in the analysis.

A retrospective review of medical charts and electronic registration was carried out between July 2013 and March 2015. Subjects who met criteria for the collection of all variables (sex, age, genotype, pancreatic insufficiency, use of vitamin supplementation and pancreatic enzymes, height, weight, lung function, number of exacerbations, 25(OH)D) were included. Anthropometric data (weight and height) and lung function were collected from medical records, in a period of 60 days before or after 25(OH)D measures.

Weight and height were routinely measured in the outpatient follow-up visits, using stadiometer and a digital scale. Anthropometric measurements were taken with the subjects wearing light clothes. The collected data were analyzed using the Anthro software (http://www.who.int/childgrowth/software/en/) and Anthro Plus (http://www.who.int/growthref/tools/en/) from World Health Organization (WHO).^(^ ^18^ ^,^ ^19^ ^)^ Data were presented in body mass index (BMI) for age and height for age (H/A) Z scores.

Spirometry was performed at the routine follow-up outpatient clinic. In this study, forced expiratory volume in 1 second (FEV_1_) and forced vital capacity (FVC) were obtained from all subjects aged over 5 years. Data were evaluated by the web tool produced by the Global Lung Function Initiative (GLI2012) and the European Respiratory Society (http://www.ers-education.org/guidelines/global-lung-function-initiative/spirometry-tools/desktop-individual-calculator.aspx) and expressed as percentage of predicted.^(^ ^20^ ^)^

Vitamin D sufficiency (25(OH)D ≥30ng/mL), deficiency (25(OH)D <20ng/mL) or insufficiency (20 to 29.9ng/mL) were defined according to the guidelines of the Cystic Fibrosis Foundation and the Endocrine Society.^(^ ^21^ ^,^ ^22^ ^)^

PE episodes were defined using the following criteria: signs and symptoms of exacerbation (fever, increased cough, change in volume or consistency of sputum, decreased appetite, weight loss and/or change in physical examination) and/or reduction in the lung function parameters of at least 5 to 10%, associated with the use of systemic antibiotics.^(^ ^11^ ^)^ The total number of exacerbations was computed in a 2-year period (previous year and year of vitamin D measurement) and the post-vitamin D dosing period (interval between measurement up to the end of the follow-up) until March 2015 (mean 5.4 months).

The present study was approved by the Scientific and Ethics Committee of *Pontifícia Universidade Católica do Rio Grande do Sul*, under number 1.143.034, CAAE: 45768115.4.0000.5336, and it was funded by *Conselho Nacional de Desenvolvimento Científico e Tecnológico* (CNPq).

### Statistical analysis

The descriptive analysis of the data was presented using proportions, mean (standard deviation) and/or median (interquartile range) depending on the characteristics of variables. χ^2^ test was used to evaluate associations between categorical variables. Spearman's test was used to evaluate the correlation between numerical variables. Comparisons of categorical and continuous outcomes between groups were analyzed by Student's *t* test and/or Mann-Whitney. Analyses were performed using Statistical Package of the Social Science (SPSS), version 17.0. Differences were considered significant with a p value <0.05.

## RESULTS

This study evaluated 37 subjects who meet all inclusion criteria and 20 were male (54*%*). Of the subjects 65*%* were aged less than 15 years and the mean age was II ±5.58 years. Most subjects with available genotype data had, at least, one F508del allele (n=17/23; 74*%*). Pancreatic insufficiency was observed in 94.6*%* of subjects, who were on pancreatic enzyme replacement therapy. All subjects received routine oral CF-specific vitamin supplementation, as recommended by the Cystic Fibrosis Foundation (birth to 12 months: 400 to 500IU/day D3; >12 months to 10 years: 800 to 1,000IU/day D3; >10 years: 800 to 2,000IU/day D3).^(^ ^21^ ^)^The mean serum 25(OH)D was 30.53±12.14ng/mL. Hypovitaminosis D (25(OH)D <30ng/mL) was observed in 54% of sample, being 40.5*%* and 13.5*%* vitamin D insufficient and deficient, respectively. Seventeen subjects (46*%*) showed 25(OH)D values within the normal range. Nutritional status was within normal ranges for most subjects (mean BMI Z-score: 0.20±1.32). Only 8*%* of the subjects had BMI Z-score lower than −2.0. In relation to H/A parameter, the mean Z-score was −0.66 ±0.95. Twenty-nine subjects performed PF tests, and the mean percentage of predicted FEV_1_ was 78.64± 27.76. Of the subjects 62%, 70% and 10*%* had respectively a greater than 70*%*, between 40 and 70% and lower than 40% predicted FEV_1_. The characteristics of the subjects are described in table 1.

**Table 1:** Baseline characteristics of the study sample (n=37)

Characteristics	
Male	20 (54)
Age, years	11±5.58
At least 1 allele F508del (n=23)	17 (74)
Pancreatic insufficiency	2 (94.6)
BMI, kg/m^2^, score Z	0.20±1.32
H/A, score Z	-0.66±0.95
FEV_1_, % of predicted (n=29)	78.64±27.76
FEV_1_ >70%	18 (62)
FEV_1_ 40-70%	8 (28)
FEV_1_ <40%	3 (10)
FVC, % of predicted (n=29)	86.20±24.66
25(OH)D, ng/mL	30.53±12.14
Vitamin D defficient, <20ng/mL	5 (13.5)
Vitamin D insufficient, (≥20 a <30ng/mL	15(40.5)
Vitamin D sufficient, ≥30ng/ml	17(46)

Values are expressed in n (%) or mean±standard deviation.

BMI: body mass index; H/A: height for age; FEV_r_ forced expiratory volume in 1 second; FVC: forced vital capacity; 25(OH)D: 25-hidroxyvitamin D.

Vitamin D levels were grouped into two classes: sufficient (25(OH)D ≥30ng/mL) and hypovitaminosis (25(OH)D <30ng/mL). The mean serum 25(OH)D was 40.27±9.82ng/mL and 22.25±6.41ng/mL in the sufficient and hypovitaminosis group, respectively. There was no association between nutritional status of subjects measured by BMI Z-score and vitamin D levels. Similar results were found in relation to age, H/A Z-scores and PF (Table 2).

**Table 2:** Comparison the characteristics and outcomes, according to vitamin D levels

Characteristics and outcomes	Vitamin D levels		p value
	Sufficient (≥30ng/mL) (n=17)	Hypovitaminosis (<30ng/mL) (n=20)	
Age, years	14 (6-16)	9.5 (5-15)	0.517
BMI, kg/m^2^, score Z	0 (-1-0.50)	0(0-1)	0.141
H/A, score Z	-1 (-1-0)	-1 (-1-0)	0.232
FVC, % of predicted	92 (69.75-110.50)	81 (73-96)	0.717
FEV_1_, % of predicted	86.50 (65.75-104.75)	72 (60-94)	0.354
PE over 2 years	2 (0.5-4.5)	4.5 (3-8)	0.007
Admission over 2 years	0 (0-1)	0 (0-1)	0.497
PE post-dosing period	0(0-1)	2 (1-2)	0.002
Admission post-dosing period	0(0-0)	0(0-0)	0.869

Values are expresses in median and interquartile range.

BMI: body mass index; H/A: height for age; FEV_r_ forced expiratory volume in one second; FVC: forced vital capacity; PE: pulmonary exacerbations.

Pulmonary exacerbations were significantly associated with lower vitamin D levels. Subjects with low vitamin D levels had more than twice exacerbations over a 2-year period (p=0.007), and significantly more exacerbations in the period after vitamin D measurements up to the end of follow up (p=0.002), as compared with subjects with vitamin D levels ≥30ng/mL. Vitamin D levels were not associated with hospital admissions.

**Figure 1:**
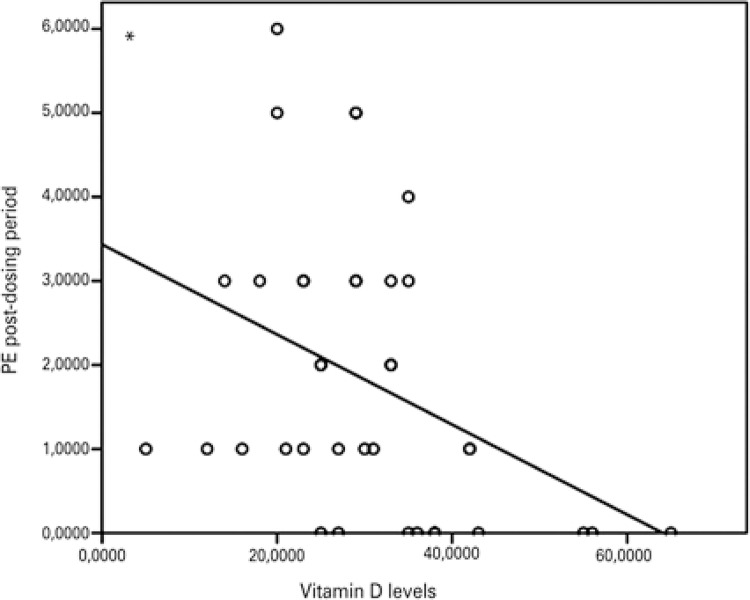
Correlation between number of exacerbations in the period post-dosing vitamin D measurement and vitamin D levels

**Figure 2:**
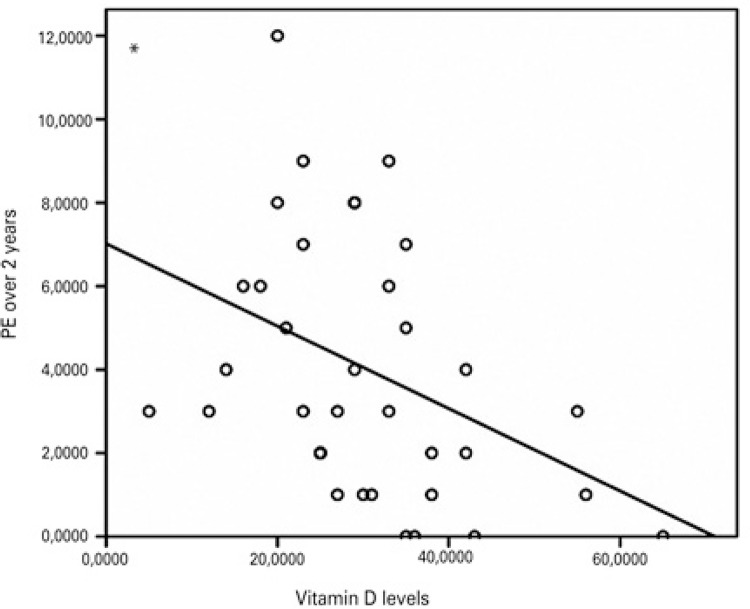
Correlation between number of exacerbations over a 2-year period and vitamin D levels

The figure 1 illustrates the correlation between the number of exacerbations in the period post-dosing of vitamin D measurements and vitamin D levels (p=0.008). The correlation between the number of PE over a 2-year period and vitamin D levels are shown in figure 2 (p=0.004).

## DISCUSSION

Vitamin D has been associated with systemic inflammation and the risk of respiratory infections in several studies.^(^ ^2^ ^,^ ^5^ ^,^ ^10^ ^,^ ^17^ ^)^ In this study, CF subjects with low vitamin D levels presented significantly more episodes of PE, which is associated with decreased lung function over time.^(^ ^11^ ^)^

Pulmonary exacerbation is one of the most important predictors of lung disease outcomes in CF patients. It has a direct impact on the quality of life of subjects, and increases morbidity and impact on healthcare costs. The frequency of PE has been associated with disease progression and decreased survival.^(^ ^11^ ^,^ ^12^ ^)^Although there is no consensus on the definition of a PE, some criteria for changes in patient's conditions are widely used in studies evaluating this outcome, such as increased cough, fever, weight loss and sputum characteristics.^(^ ^11^ ^,^ ^23^ ^)^ A PE diagnosis often triggers the decision of systemic antibiotic treatment. In this study, we used clinical changes and antibiotic prescription as the main criteria for the definition of PE.

Vitamin D deficiency has been recognized as a possible factor associated with exacerbation of symptoms in CF patients, probably impairing their baseline situation.^(^ ^6^ ^,^ ^7^ ^,^ ^10^ ^,^ ^17^ ^)^ Pulmonary exacerbations were clearly associated with the levels of vitamin D in the present study. In a 2-year period, subjects with 25(OH)D <30ng/mL had significantly more episodes of exacerbations than subjects with sufficient vitamin D levels. There are few studies that linked vitamin D levels and PE in children with CF. In two of them, lower levels of 25(OH)D were also associated with higher rates of PE.^(^ ^24^ ^,^ ^25^ ^)^ McCauley et al., however, defined PE as hospital admission only.^(^ ^24^ ^)^ In a recent study, unlike the two previously mentioned, Butenko et al., showed no effect of vitamin D levels on PE rates.^(^ ^26^ ^)^ However, this study included a smaller number of subjects (n=27). Hospital admissions were not associated with vitamin D levels in the present study, possibly due to the limited sample size and/or because hospitalizations are not as frequent as PE.

It is uncertain whether the increase of vitamin D levels in subjects with CF would promote an improvement of pulmonary condition. A pilot study involving supplementation with high dose of cholecalciferol (vitamin D3) or placebo at the moment adults were admitted for PE, observed a trend for better clinical outcomes, as well as antibiotic therapy-free days in a period of 6 months.^(^ ^27^ ^)^ More intervention studies, however, would be required for a definitive confirmation of causal associations.

The possible immunomodulatory effects of vitamin D reported in several studies could explain its likely role in the prevention of PE.^(^ ^1^ ^,^ ^2^ ^,^ ^28^ ^)^ Grossmann et al., showed that high-dose vitamin D supplementation was associated with a reduction in two inflammatory cytokines: interleukin 6 and tumor necrosis factor alpha.^(^ ^29^ ^)^A large Scandinavian study with CF patients found a significant inverse correlation between 25(OH)D and immunoglobulin G.^(^ ^17^ ^)^ Herscovitch et al., concluded that supplementation with vitamin D may be used to modulate the immune and inflammatory response in CF subjects. Nonetheless, greater doses than those routinely used might be required for this purpose.^(^ ^5^ ^)^The current classification of 25(OH)D levels was based on values considered optimal for maintaining bone health.^(^ ^22^ ^)^ Therefore, higher levels might be necessary to boost extra skeletal vitamin D functions. A dose of appropriate supplementation, regardless of the purpose, is uncertain. Other studies also reported that subjects with CF may require additional doses of vitamin D to maintain 25(OH)D values above 30ng/mL.^(^ ^27^ ^,^ ^30^ ^)^ This may explain the high frequency of vitamin D deficiency, even in subjects using routine oral supplementation. In the present study, all children and adolescents with CF were prescribed routine vitamin supplementation. Despite this, more than half of the subjects had suboptimal levels of vitamin D. Previous cross-sectional study in pediatric subjects with CF described a prevalence of vitamin D deficiency even higher (95%), using the same cutoff level.^(^ ^15^ ^)^ Two other studies in CF children and adolescents found a prevalence of suboptimal vitamin D levels of 7*%* and 37*%* of sample.^(^ ^13^ ^,^ ^16^ ^)^ However, the cutoff points were lower than currently recommended (<25nmol/L or 10 ng/mL and <50nmol/L or 20ng/mL, respectively).

The present study found no correlation between serum levels of 25(OH)D and lung function. Although studies in adults with CF demonstrated an association between low levels of 25(OH)D and reduced lung function,^(^ ^7^ ^,^ ^17^ ^)^ other studies in children did not find such association.^(^ ^14^ ^,^ ^16^ ^)^ The population of our study did not have severe *deficits* in lung function, which might suggest that the impact of hypovitaminosis D in FEV_1_ and FVC is greater with the progression of disease. Similar explanation may help to understand the lack of association between vitamin D and BMI and H/A.

This study had some limitations. First, the retrospective nature can limit conclusions, but further prospective studies may help to confirm or reject the results of the present study. The sample size was relatively small but similar to previous studies that investigated PE as a clinical outcome.

In the present study, hypovitaminosis D was associated with higher rates of PE in children and adolescents with CF. Considering the current evidence on the important impact of vitamin D on lung health, inflammation and immunity, it is essential to screen for hypovitaminosis D in CF patients. Using higher doses of vitamin D as adjunctive therapeutic to slow disease progression looks promising. However, further randomized controlled trials are required to prove this possible benefit.

## CONCLUSION

In this sample of children and adolescents with cystic fibrosis hypovitaminosis D was associated with higher rates of pulmonary exacerbations. It is possible that hypovitaminosis D may be indicated as a marker of disease severity in cystic fibrosis, since it is a possible factor associated with exacerbation of symptoms and worsening of the underlying situation. Further prospective and randomized studies are necessary to investigate causality of this association.
